# Novel biomarkers for risk stratification of Barrett’s oesophagus associated neoplastic progression–epithelial HMGB1 expression and stromal lymphocytic phenotype

**DOI:** 10.1038/s41416-019-0685-1

**Published:** 2019-12-13

**Authors:** Ross J. Porter, Graeme I. Murray, Daniel P. Brice, Russell D. Petty, Mairi H. McLean

**Affiliations:** 1 School of Medicine, Medical Sciences and Nutrition, University of Aberdeen, Foresterhill, Aberdeen, AB25 2ZD UK; 2 Division of Molecular and Clinical Medicine, School of Medicine, University of Dundee, Dundee, DD1 1GZ UK

**Keywords:** Mucosal immunology, Mechanisms of disease

## Abstract

**Background:**

The incidence of oesophageal adenocarcinoma is increasing globally. Barrett’s oesophagus (BO) is a pre-malignant condition with no biomarker to risk stratify those at highest risk of dysplasia and malignant transformation.

**Methods:**

Subcellular epithelial protein (HMGB1, p53, RUNX3) expression, alongside expression of CD20, CD4, CD8 and Foxp3 to characterise stromal B lymphocyte, and helper, cytotoxic and regulatory T-lymphocyte cell infiltrate, respectively, was assessed by immunohistochemistry in 218 human tissue samples including normal oesophageal/gastric biopsies (*n* = 39), BO (non-dysplasia, dysplasia, non-dysplastic background from progressors to dysplasia or cancer, *n* = 121) and oesophageal adenocarcinoma (*n* = 58).

**Results:**

There is a dynamic subcellular epithelial expression of HMGB1 (loss of nuclear, emergence of cytoplasmic), associated with epithelial p53 expression and differential immune cell phenotype in oesophageal neoplastic progression. We identify a protein signature and lymphocyte infiltrate in non-dysplastic BO when progressive disease (dysplasia or adenocarcinoma) is present but not histologically represented in the biopsied field. There is a dynamic stromal lymphocytic infiltrate in oesophageal neoplastic progression.

**Conclusions:**

This data reveals novel insights into the microenvironment of BO and progression towards cancer and identifies a novel high-risk biomarker of disease progression to aid surveillance strategies to identify early progression and impact future incidence of oesophageal cancer.

## Background

The global incidence of oesophageal cancer is increasing with over half a million cases worldwide in 2018, accounting for 1 in 20 of all cancer deaths. In the UK,^1^ there are 9,000 new cases per year with a 15% 5-year survival.^2^ Barrett’s oesophagus (BO) is a pre-malignant condition for oesophageal adenocarcinoma that affects 1.6–8% of the UK population.^3^ BO is defined by histological evidence of epithelial metaplasia from normal stratified squamous epithelium to mucin-secreting columnar epithelium in the distal oesophagus. These metaplastic cells can undergo further transformation to dysplasia or malignancy. Although the lifetime risk of transformation remains under debate, a previous UK study of nearly 8000 patients suggested this could be as high as 1 in 14 patients, although a risk of ~0.5% per annum has been more widely reported.^4^ The annual incidence of oesophageal adenocarcinoma is 0.33% and 1.40% in non-dysplastic and dysplastic BO, respectfully.^5,6^ Cancer incidence has been estimated to be 40-times greater in high grade dysplasia, compared with the general population.^7^ Currently, it is not possible to predict those with BO at high risk of progression to dysplasia and malignancy. Therefore, all patients are offered interval endoscopic surveillance with biopsy to detect cell dysplasia or early cancer amenable to endoscopic therapy.^8^ This strategy carries significant resource implications and subjects individuals to repeated invasive investigation. There is clearly a clinical need to identify those at highest risk of progression at the point of diagnosis and to focus endoscopic and clinical resource accordingly. Currently, the pathogenesis of metaplastic-dysplastic-malignant progression is not fully understood.^3,9^ However, there have been a variety of proposed biomarkers to aid this risk stratification^10^, including transcriptional changes,^11^ tissue microRNAs^12,13^ or circulating glycoproteins^14^ and breath volatiles.^15^

Identifying novel cellular mechanisms that underlie BO pathogenesis and progression would identify biomarkers of risk, inform less invasive and more sensitive monitoring strategies and, by detecting progressive disease early, impact incidence and prognosis of oesophageal cancer.

With this clinical problem in mind, our aim was to define the role of the protein high mobility group box-1 (HMGB1) in BO and progression to cancer. Due to its cellular functions, and link to key protein targets, we hypothesised that HMGB1 is important in the pathogenesis of BO to cancer. HMGB1 is a ubiquitous nuclear protein that binds to the minor grove of DNA to stabilise the genome and regulate gene expression.^16^ Cellular stress results in phosphorylation of HMGB1, inducing cytoplasmic and extracellular shuttling.^17^ Once extracellular, HMGB1 influences epithelial cell behaviour and immune cell responses.^18,19^ HMGB1 is differentially expressed in malignancy at a number of body sites, including liver, stomach, colon, bladder, pancreas, prostate and cervix. In squamous oesophageal cancer, HMGB1 promotes lymphangiogenesis by regulating expression of VEGF-C and VEGF-D, and is negatively correlated to survival.^20^ There is no data on expression of HMGB1 in BO or oesophageal adenocarcinoma.

We propose that identifying important effector proteins, downstream of HMGB1, is key to fully characterising the role of HMGB1 in pre-malignant and malignant pathologies. p53 is a pivotal tumour suppressor protein that becomes dysregulated in various cancers.^21^ In oesophageal adenocarcinoma, ~75% of patients have p53 mutations and consequently express strong nuclear p53.^22^ In BO, only foci of dysplasia exhibit p53. Some centres incorporate p53 expression as a diagnostic aid for identifying dysplasia.^8^ Of interest here, HMGB1 can facilitate p53-DNA binding, induce a p53-dependent senescent growth arrest, and complex with p53 to mediate autophagy and apoptosis; localisation and expression of each protein influences the other.^23–25^ We hypothesise that loss of nuclear HMGB1 expression could impact p53 expression in oesophageal neoplastic progression.

Similarly, runt-related transcription factor 3 (RUNX3) is a highly-conserved transcription factor important in the activation, proliferation and differentiation of lymphocytes.^26^ Promoter hypermethylation of *RUNX3* is associated with an increased risk of progression, as well as poorer survival in oesophageal cancer.^27^ Hypermethylation of *RUNX3* occurs as an early event in BO and is an independent risk factor for progression to dysplasia or oesophageal adenocarcinoma.^28^ HMGB1 has been identified as a potent inducer of an interferon-γ producing T_H_17 lymphocyte immune cell response, via regulation of transcription factors T-bet and RUNX3, leading to progression of atherosclerosis.^29^ Therefore, we hypothesise that RUNX3 may be an important downstream mediator influenced by HMGB1 expression.

HMGB1 also co-ordinates immune cell function, although this is not well characterised.^30^ The mechanisms by which extracellular HMGB1 influences immune activity is complex and dependant on post-translation modification status through direct interaction with transmembrane receptors such as RAGE or TLR-4, or via complex formation with co-factors, such as binding to CXCR4 as a heterodimer with CXCL12, or binding to TLR9 via a complex with CpG-ODN.^31^ It now known that there are many mechanisms directing HMGB1 induced immune responses. It is universally recognised that stromal immune responses are an important aspect of carcinogenesis and one of the hallmarks of cancer.^32^ Despite this, to date there has been no exploration of the dynamic inflammatory cell microenvironment in BO.

The aim of this study was to define the expression of HMGB1, key downstream proteins and lymphocyte phenotype in oesophageal neoplastic progression from BO to dysplasia to oesophageal adenocarcinoma in human tissue samples.

Here, by demonstrating emergence of cytoplasmic HMGB1, nuclear p53 and nuclear RUNX3 expression in oesophageal neoplastic progression, alongside a dynamic inflammatory cell infiltrate adjacent to BO mucosa, we demonstrate novel mechanistic insights into the pathogenesis of BO and malignant transformation. Notably, we have identified a protein signature strongly associated with presence of progressive disease at time of sampling, even although the dysplastic or carcinomatous mucosa is not apparent in the endoscopic biopsies obtained, and therefore identifying high risk individuals that need further assessment. These data from this discovery cohort offers high translational potential as a novel biomarker to predict presence of disease progression not histologically sampled by random biopsies, offers a new biomarker to aid diagnosis of dysplasia, and uncovers a novel target pathway to develop treatment strategies to deter malignant transformation in BO.

## Methods

### Tissue specimens

**Fig. 1:**
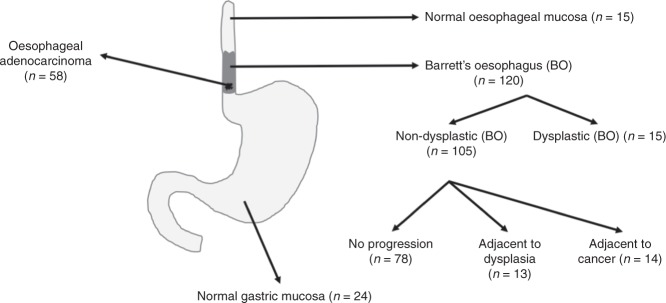
Human oesophageal tissue specimens were sourced from the Grampian Tissue Biorepository (*n* = 218), including 58 oesophageal adenocarcinoma, 39 biopsies of normal mucosa (15 oesophageal, 24 gastric), 106 non-dysplastic Barrett’s oesophagus (BO) biopsies (78 from patients with no evidence of dysplasia or cancer, 14 from patients with an adjacent focus of dysplasia and 14 adjacent BO from patients with adenocarcinoma) and 15 dysplastic BO biopsies.

Formalin-fixed paraffin-embedded tissue was sourced from the Grampian Biorepository (*n* = 218 total, Fig. 1). Tissue cores within a pre-published tissue microarray^33^ representing 58 oesophageal adenocarcinomas, 15 normal oesophageal mucosa, 24 normal gastric mucosa and 14 BO mucosa adjacent to oesophageal adenocarcinoma were assessed. In addition, 107 endoscopically retrieved biopsy specimens with a histological diagnosis of BO were analysed; 78 endoscopically retrieved biopsies of BO from patients who had not progressed, 15 endoscopically retrieved biopsies displaying low grade dysplastic BO, 14 endoscopically retrieved biopsies of non-dysplastic BO adjacent to an area of dysplasia. On average, three biopsies were retrieved per patient. In total, 121 tissue samples of BO were included in the analysis. The histological diagnosis of each tissue was confirmed by an expert gastrointestinal pathologist (G.I.M.).

### Tissue microarray

Tissue cores were obtained at time of surgical resection for oesophageal or gastric cancer between 2004 and 2010 at Aberdeen Royal Infirmary as previously published.^33^ Supplementary Table 1 describes clinico-pathological parameters and their relationship with overall survival and validates the TMA as representative of pathology.

### Immunohistochemistry

Intensity of epithelial nuclear and cytoplasmic expression of target proteins (HMGB1, p53 and RUNX3) were each assessed immunohistochemically in all tissue specimens (*n* = 218). Stromal inflammatory cell phenotype was assessed in BO (*n* = 121). Expression of CD20, CD4, CD8 and Foxp3 were used to identify B lymphocytes, and helper, cytotoxic and regulatory T-lymphocyte cell subsets, respectively. Antibody characteristics, dilutions, positive controls and methods of antigen-retrieval are outlined in Supplementary Table 2. In total, 1294 stained tissue sections were analysed.

Four micrometre serial tissue sections were cut and placed onto 3-aminopropyltriethoxysilane-coated slides for immunohistochemical analysis. Specimens were dewaxed in xylene, rehydrated in alcohol and subject to heat-mediated antigen retrieval by microwaving at 800 W for 20 min in either 10 mM citrate (pH6) or EDTA (pH 7.8) buffer. Immunohistochemistry was performed using the Dako Autostainer and Dako EnVision+^™^ peroxidase-linked, biotin-free synthesis (Dako, Ely, UK) with 3′-3′-diaminobenzidine as chromogen.^34,35^ Positive and negative (exclusion of primary antibody) controls were included within each experiment.

### Evaluation of immunostaining

#### Epithelial proteins (HMGB1, p53, RUNX3)

All stained specimens were independently assessed under light microscopy by two observers (R.J.P. and D.P.B. or M.H.M.). Epithelial intensity and location of HMGB1, p53 and RUNX3 expression was assessed using a semi-quantitative, previously published scoring methodology of absent, weak, moderate or strong immunopositivity in nuclear and cytoplasmic compartments.^34,36^ The intensity score within the area of strongest immunopositivity per sample was independently recorded by observers. Discordant scores were reviewed and resolved by discussion. An expert gastrointestinal pathologist (GIM) reviewed and discussed specimens that remained unresolved. Observers scored blind to clinico-pathological data.

#### Stromal Immune cell phenotype

The number of CD20^+^, CD4^+^, CD8^+^ and Foxp3^+^ stromal lymphocytes were assessed as previously published^37^ in normal oesophageal and gastric mucosa (representing normal squamous and normal glandular epithelium, respectively) versus BO tissue samples. The number of positively stained lymphocytes were counted in one high-power field at magnification X40 within the area of most positive lymphocyte infiltration, immediately adjacent to the appropriate histological epithelial compartment.

### Statistical analysis

All statistical tests were performed using IBM® SPSS® Statistics (Version 24.0.0.0) and a two-tailed alpha was set at 0.05. 95% confidence intervals are included where appropriate. The association of epithelial HMGB1, p53 and RUNX3 expression with tissue histology or clinico-pathological parameters was evaluated using *χ*^2^ and Fisher’s exact tests. Associations between protein expression or clinicopathological data and survival was assessed using Kaplan–Meier survival analysis, log-rank tests and Cox-regression analysis. Mann–Whitney U tests and Kruskal-Wallis test with Bonferroni correction was used to assess for associations between lymphocyte populations and histological cell types in normal and BO specimens. Samples were analysed as absent and weak versus moderate and  strong immunopositivity. To ensure comprehensive assessment, three further analysis methodologies were used (immunonegativity versus immunopositivity; absent versus weak versus moderate versus strong; and strong versus  all other intensities), as previously published (Supplementary Tables 3–5).^36,38^ Results presented in the paper represent absent and weak versus moderate and strong intensity expression comparisons, and refer to supplementary methodologies when appropriate. The number of specimens per analysis varied and are declared throughout. This was due to the finite nature of paraffin block tissue, incorrect tissue type on the slide or absent or folded specimen.

## Results

**Fig. 2:**
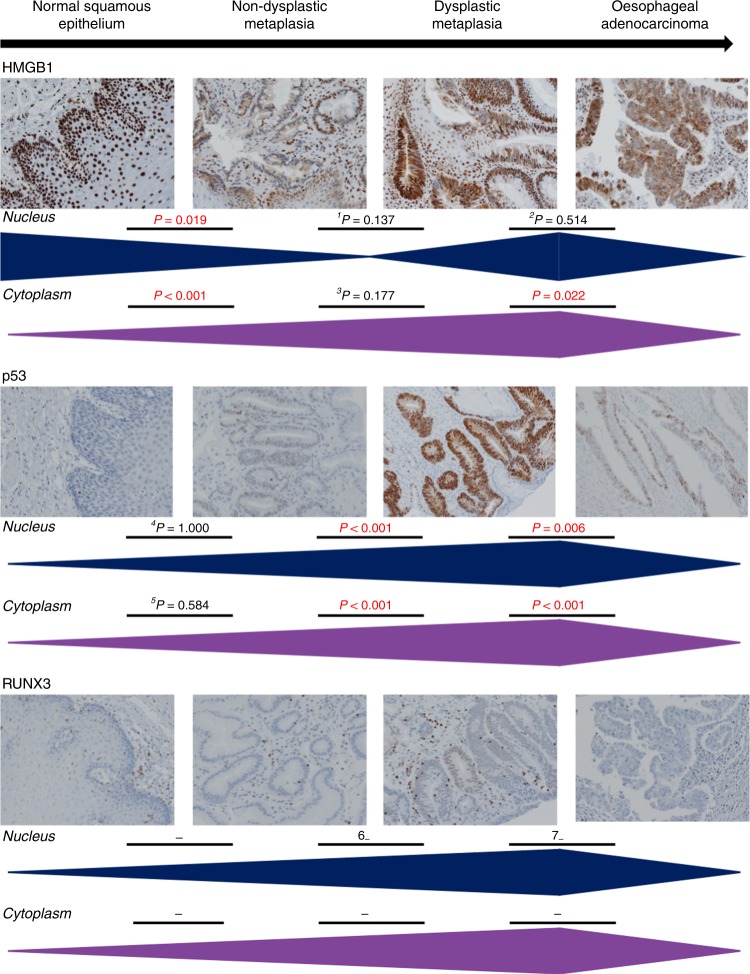
Dynamic subcellular expression of HMGB1, p53 and RUNX3 is associated with oesophageal neoplastic progression. Representative photomicrographs across each stage of oesophageal neoplastic progression demonstrating HMGB1, p53 and RUNX3 expression profile. Bars below photomicrographs represent the trend in expression pattern throughout oesophageal neoplastic progression. Associations were analysed by *χ*^2^ and Fisher’s exact test and *p* values represent absent + weak vs. moderate + strong protein expression intensity. Extended analysis revealed ^1^*p* ≤ 0.008, ^2^*p* ≤ 0.008, ^3^*p* ≤ 0.002, ^4^*p* = 0.003, ^5^*p* ≤ 0.004, ^6^*p* = 0.001 and ^7^*p* ≤ 0.002, and are detailed in Supplementary Tables 3–5.

**Fig. 3:**
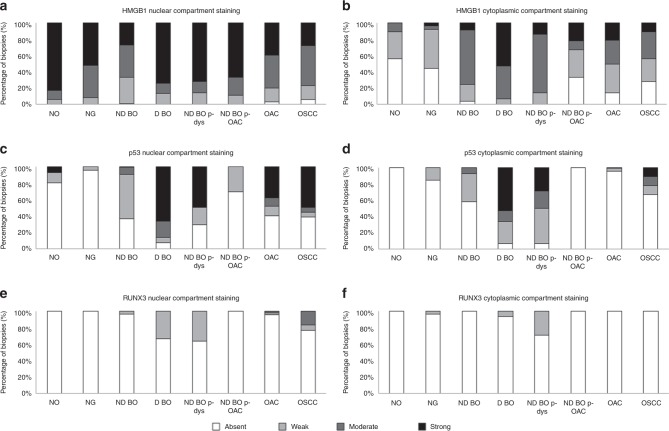
Frequency distribution analysis reveals dynamic subcellular expression of HMGB1, p53 and RUNX3 is associated with oesophageal neoplastic progression. Intensity expression of **a** epithelial nuclear HMGB1, **b** epithelial cytoplasmic HMGB1, **c** epithelial nuclear p53, **d** epithelial cytoplasmic p53, **e** epithelial nuclear RUNX3 and **f** epithelial cytoplasmic RUNX3, in oesophageal neoplastic progression. Graphs illustrate the frequency distribution of absent, weak, moderate and strong intensity of immunopositivity in NO–normal oesophagus, NG–normal gastric, ND BO–non-dysplastic BO, D BO–dysplastic BO, ND BO p-dys–non-dysplastic BO in patients with dysplasia, ND BO p-OAC–non-dysplastic BO in patients with oesophageal adenocarcinoma, OAC–oesophageal adenocarcinoma. Results of statistical analysis of this data can be viewed in Supplementary Tables 3–5.

Representative photomicrographs of epithelial target protein expression and frequency distribution of intensity expression are reported in Fig. 2, Supplementary Fig. 1 A–G, and Fig. 3, respectively. Statistical comparative analyses are detailed in Table 1 and Supplementary Tables 3–5.

**Table 1:** Association between epithelial target protein expression and histological diagnosis.

Comparisons	HMGB1		p53		RUNX3	
	Nucleus	Cytoplasm	Nucleus	Cytoplasm	Nucleus	Cytoplasm
*Pre-malignant*						
NO v NG	1	1	0.375	a	^a^	^a^
NO v ND BO	**0.019**	**<0.001**	1	0.584	^a^	^a^
NO v D BO	0.579	**<0.001**	**<0.001**	**<0.001**	^a^	^a^
NO v ND BO p-dys	0.568	**<0.001**	**0.014**	**0.002**	^a^	^a^
NO v ND BO p-OAC	1	0.295	1	a	^a^	^a^
NG v ND BO	**0.018**	**<0.001**	0.187	0.333	^a^	^a^
NG v D BO	0.622	**<0.001**	**<0.001**	**<0.001**	^a^	^a^
NG v ND BO p-dys	0.609	**<0.001**	**<0.001**	**<0.001**	^a^	^a^
NG v ND BO p-OAC	1	0.102	a	a	^a^	^a^
ND BO v D BO	0.137	0.177	**<0.001**	**<0.001**	^a^	^a^
ND BO v ND BO p-dys	0.211	0.509	**0.001**	**0.001**	^a^	^a^
ND BO v ND BO p-OAC	0.261	**0.015**	0.588	0.585	^a^	^a^
D BO v ND BO p-dys	1	0.598	**0.05**	0.462	^a^	^a^
D BO v ND BO p-OAC	1	**0.004**	**<0.001**	**<0.001**	^a^	^a^
ND BO p-dys v ND BO p-OAC	1	**0.023**	**0.006**	**0.006**	^a^	^a^
*Oesophageal adenocarcinoma*						
OAC v NO	0.116	**0.002**	**0.002**	1	1	^a^
OAC v NG	0.099	**<0.001**	**<0.001**	1	1	^a^
OAC v ND BO	0.232	**0.001**	**<0.001**	**0.013**	0.294	^a^
OAC v D BO	0.514	**0.002**	**0.006**	**<0.001**	1	^a^
OAC v ND BO p-dys	0.515	**0.019**	1	**<0.001**	1	^a^
OAC v ND BO p-OAC	0.682	0.49	**0.001**	1	1	^a^

Dynamic subcellular expression of HMGB1, p53 and RUNX3 is associated with oesophageal neoplastic progression. Numerical values represent *p* value from analysis of absent + weak vs. moderate + strong expression comparisons (***χ***^2^ and Fisher’s exact test)

Significant values denoted in bold

*NO* normal oesophagus, *NG* normal gastric, *ND BO* non-dysplastic BO, *D BO* dysplastic BO, *ND BO p-dys* non-dysplastic BO in patients with dysplasia, *ND BO p-OAC* non-dysplastic BO in patients with oesophageal adenocarcinoma, *OAC* oesophageal adenocarcinoma

^a^no expression

### Dynamic subcellular expression of HMGB1 is associated with oesophageal neoplastic progression

As expected from its known biology, HMGB1 was strongly expressed in the nuclei of normal oesophageal epithelium. This expression was reduced in intensity upon metaplastic change to non-dysplastic BO (*p* = 0.019). In contrast, HMGB1 was not expressed or expressed weakly in the cytoplasm of normal oesophageal epithelium. However, cytoplasmic expression increased in intensity in both non-dysplastic and dysplastic BO (both *p* < 0.001) and remained present in oesophageal adenocarcinoma, although weaker in intensity to non-dysplastic BO (*p* = 0.001) and dysplastic BO (*p* = 0.002). Oesophageal adenocarcinoma expressed stronger cytoplasmic HMGB1 compared with normal epithelium (*p* = 0.002).

On extended analysis, nuclear HMGB1 expression intensified (*p* ≤ 0.008) and cytoplasmic expression intensity increased further in foci of dysplasia (*p* ≤ 0.002) compared with non-dysplastic BO (Supplementary Table 3).

### HMGB1 expression intensity in BO indicates presence of histologically distinct progressive oesophageal neoplasia

There was an increased intensity of nuclear HMGB1 in the background BO in those that had progressed to either dysplasia (71%) or cancer (67%) compared with BO from non-progressors (27%), *p* ≤ 0.017 and *p* = 0.024, respectively (Supplementary Table 3).

In addition, patients who had progressed to cancer also expressed weaker epithelial cytoplasmic HMGB1 in their background BO (absent + weak intensity in 67%) compared with patients who did not have malignancy (absent + weak intensity in 24%), *p* = 0.015. Cytoplasmic expression of HMGB1 was similar in background BO whether dysplasia was present or not.

Therefore, this data reveals a subcellular dynamic localisation and change in expression intensity of HMGB1 in oesophageal neoplastic progression. These changes were demonstrable in background BO when dysplasia or cancer was present outside the histologically sampled mucosa. In light of this finding, we then explored the biological cellular consequences of this HMGB1 expression signature with initial focus on expression of key downstream effector protein expression.

### Epithelial nuclear and cytoplasmic p53 expression is associated with oesophageal neoplastic progression

Nuclear p53 was absent in the majority (80%) of normal epithelium and emerged in dysplastic BO (87% as moderate + strong expression), *p* < 0.001, as expected. Oesophageal adenocarcinoma expressed stronger nuclear p53 compared with normal mucosa (*p* = 0.002) and non-dysplastic BO (*p* < 0.001), and weaker nuclear p53 expression compared with dysplastic BO (*p* = 0.006). This expression pattern was similar for p53 within the cytoplasmic cellular compartment. Notably, as identified in HMGB1 expression analysis, p53 expression was associated with presence of neoplastic progression even although this was not histologically present in the endoscopically sampled mucosa; patients who had progressed to dysplasia expressed stronger nuclear p53 in their background BO epithelia (50% moderate + strong intensity), compared with patients who had not progressed (9% moderate + strong intensity), *p* = 0.001.

### Weak intensity of epithelial nuclear RUNX3 emerges in dysplastic BO

RUNX3 was not expressed in normal epithelium, and rarely (<3%) in non-dysplastic BO or oesophageal adenocarcinoma epithelium. There was not a spectrum of intensity profiles to allow our focussed absent + weak intensity versus moderate + strong intensity analysis. Therefore, we employed extended comparison methods (Supplementary Table 5) to assess presence versus absence and there was emergence of weak intensity of epithelial nuclear RUNX3 in dysplastic BO, compared with normal mucosa (*p* = 0.013), non-dysplastic BO (*p* = 0.001) and oesophageal adenocarcinoma (*p* = 0.002). Patients who had progressed to dysplasia also expressed weak RUNX3 in their background BO compared with non-dysplastic BO in patients who had not progressed (*p* = 0.001). These data highlights differential expression of this protein but the weak intensity is not discriminatory for extrapolation of this protein to a viable biomarker to aid diagnosis and risk stratification.

### Association between HMGB1 and p53 expression in oesophageal neoplastic progression

The literature supports direct interaction between HMGB1 and p53, and HMGB1 and RUNX3 as discussed previously. Here, we demonstrate significant association between cellular compartment expression patterns of HMGB1, p53 and RUNX3 in oesophageal neoplastic progression (Supplementary Tables 6).

### Stromal lymphocytic phenotype

**Fig. 4:**
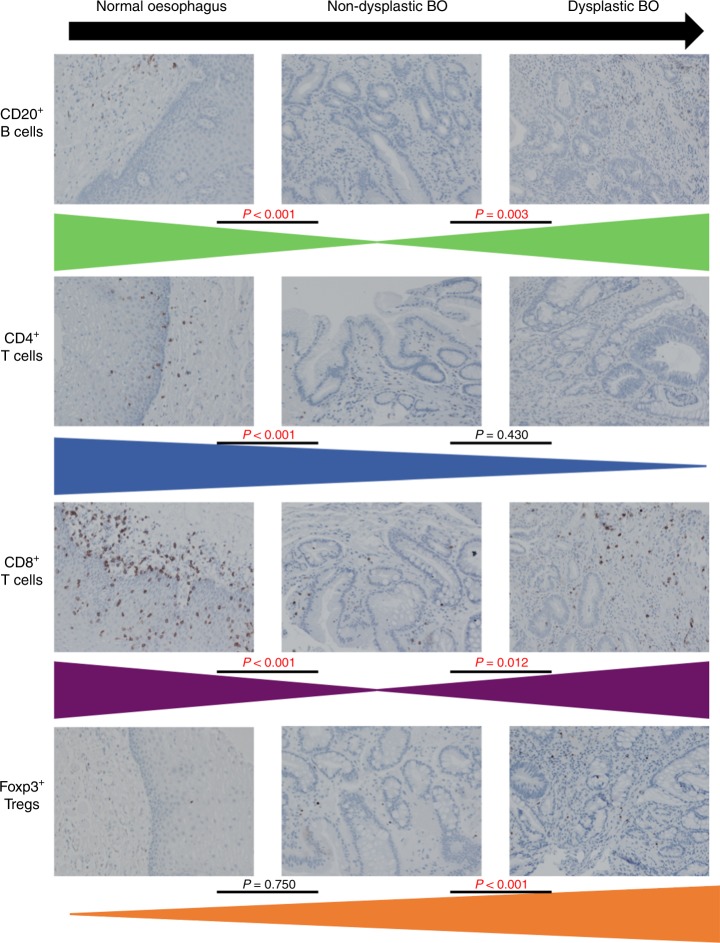
Oesophageal neoplastic progression is associated with a dynamic stromal lymphocyte phenotype. Representative photomicrographs across each stage of oesophageal neoplastic progression, representing CD20+ B lymphocytes, CD4^+^ Th cells, CD8^+^ cytotoxic T cells and Foxp3^+^ regulatory T cells. Bars below photomicrographs represent the trend in respective lymphocyte numbers throughout neoplastic progression of BO. Analysis by Mann–Whitney *U* test.

**Fig. 5:**
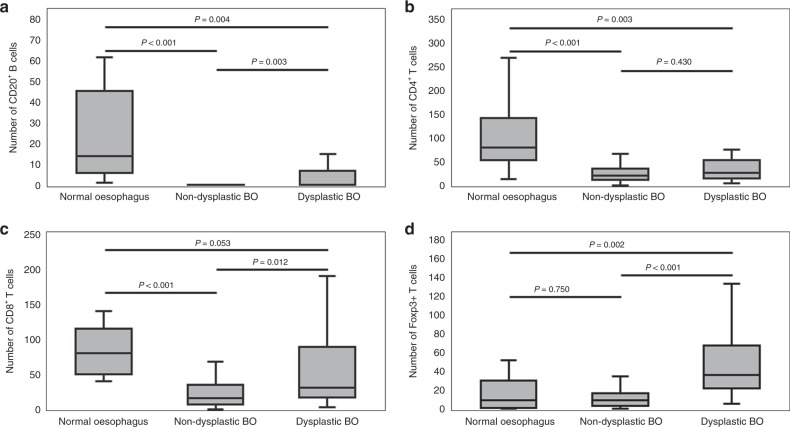
Immunophenotype of Barrett’s oesophagus stromal microenvironment is dynamic and associated with epithelial HMGB1 expression. Boxplots describing the median, 5th and 95th percentiles for **a** CD20^+^ B-cells, **b** CD4^+^ T-cells, **c** CD8^+^ T-cells and **d** FoxP3^+^ T-cells (Tregs) in Barrett’s oesophagus. Analysis by Mann–Whitney *U* test or Kruskal-Wallis Test with Bonferroni correction.

We were struck by the dramatic loss of nuclear and emergence of cytoplasmic HMGB1 in metaplastic transformation of normal epithelium to BO. To further characterise the potential biological consequence of this, we defined the surrounding stromal immune cell phenotype in normal and BO epithelium (Figs. 4, 5, Supplementary Fig. 1I–L and Supplementary Tables 7 and 8). HMGB1 was strongly expressed in all lymphocytes across all stages of oesophageal neoplastic progression (Supplementary Fig. 1H).

### Changes in stromal lymphocyte phenotype is associated with BO and dysplasia

Compared with normal epithelium, non-dysplastic BO is associated with reduced lymphocytic infiltration of CD20^+^ B-cells (*p* < 0.001), CD4^+^ T-cells (*p* < 0.001) and CD8^+^ T-cells (*p* < 0.001). In areas of dysplastic BO there is an increase of CD20^+^ B-cells (*p* = 0.003) and CD8^+^ T-cells (*p* = 0.012) and an increase in Foxp3^+^ Tregs (*p* < 0.001) compared with non-dysplastic BO. Individuals with BO who progressed to dysplasia demonstrated an immune cell infiltrate signature in background non-dysplastic BO characterised by increased CD20 + B-cells (*p* = 0.038) compared with non-dysplastic BO of non-progressors. Similarly, patients progressed to adenocarcinoma displayed increased CD20^+^ (*p* < 0.001), CD4^+^ (*p* = 0.003) and CD8^+^ (*p* = 0.014) lymphocytes in the background non-dysplastic BO compared with non-progressors.

## Discussion

This data reveals novel insights into the cellular microenvironment of Barrett’s oesophagus and oesophageal neoplastic progression, defined as dynamic subcellular epithelial expression of HMGB1, associated with epithelial p53 expression and a differential adjacent immune cell phenotype.

We demonstrate that changes in the expression intensity and location of HMGB1 are particularly relevant in pre-malignant oesophageal pathology and progression towards malignancy, rather than offering a discriminatory biomarker in cancer itself. This is suited to use as a prognostic biomarker for risk stratification in BO and to allow better resource distribution and fits into the current clinical gap.

There are emerging novel technologies in this field for diagnosis and surveillance of BO. An example currently in clinical trial is the Cytosponge^TM^ to obtain oesophageal cells for cytology analysis with TFF3 biomarker expression^39,40^ and gene methylation status.^41^ Our data demonstrates that cytoplasmic HMGB1 is identified in metaplastic and not normal oesophageal or gastric mucosa, and therefore there is potential scope for HMGB1 expression to enhance diagnostic accuracy of these emerging technologies.

Our data reveals that HMGB1 expression in background Barrett’s mucosa can predict the presence of dysplasia or cancer in histologically distinct mucosa, despite the dysplastic or carcinomatous epithelium being absent from the endoscopically sampled tissue. This raises the potential for immunohistochemical detection of HMGB1 expression pattern to be used clinically in BO as a biomarker of likely focal progression even when biopsies do not include a focus of dysplasia or cancer. A strong nuclear HMGB1 in BO epithelium in random sampling biopsies should raise suspicion of progression to dysplasia or cancer even although that progressive neoplastic focus has not been sampled. To our knowledge, there are currently no alternative risk stratification tools in background sampled BO to detect synchronous progression in clinical practice. This novel risk stratification could potentially impact clinical management in three ways.

Firstly, this HMGB1 expression signature could facilitate a focussed specialist endoscopic resource to those at perceived greater risk of disease progression, with timely specialist endoscopic re-assessment and re-biopsy.

Secondly, could this risk expression profile direct specific endoscopic therapy in the absence of sampled dysplasia or cancer to reduce future risk of malignancy? There has been debate for some time in the literature for endoscopic ablation in BO without dysplasia as a cancer prevention strategy^42^ but this is not thought cost effective^43^ and has implications for risk exposure (radiofrequency ablation (RFA) is associated with stricture 5%, bleeding 1% and perforation 0.6% in a meta-analysis)^44^ in a patient population with low risk of progression. Therefore, an endoscopic ablation strategy for all BO remains controversial and is not recommended in clinical practice.^45^ Nevertheless, current endoscopic surveillance with visible lesion plus quadrantic biopsies remains problematic due to a variety of factors such as variability in endoscopist expertise at recognising lesions, sufficient representative material for pathologists in biopsies, and adherence to recommended biopsy protocol with regard to number and quadrantic approach.^45^ Therefore, an alternative means to risk stratify individuals is needed. As an example, Das et al. incorporated previously identified gene mutations associated with progression risk to denote mutational load and applied Markov modelling in a hypothetical cohort to predict those at high risk for endoscopic ablation.^46^ Through cost effectiveness analysis, they demonstrated that this approach, to direct RFA to a biomarker-identified high risk group, was superior to other surveillance strategies including current clinical guidelines. There may be potential for our HMGB1 expression profile, easily assessed by widely available and affordable modality of immunohistochemistry, to be applied in this capacity. However, our data suggests that strong intensity of HMGB1 in background BO is not apparent in all cases. Therefore, this approach requires further assessment of risk and cost effectiveness.

Thirdly, current guidelines initially suggested endoscopic ablation therapy in patients with BO and histological evidence of flat high grade dysplasia,^8^ with amendment recently as new evidence has emerged to support endoscopic ablation with RFA in patients with histologically confirmed low grade dysplasia (LGD).^47^ These updated guidelines were informed by a randomised controlled multi-centre study where 68 patients with LGD-BO were treated with either RFA or standard endoscopic surveillance for progression. There was a progression rate to high grade dysplasia or cancer of 1% vs 26.5% in the RFA treated versus surveillance group over 3 years, respectively. RFA treatment in these patients was well tolerated and with low risk of adverse events. The greatest risk was post-RFA stricture in 12% of patients, requiring endoscopic dilatation.^48^ There are difficulties and controversies in the histologically diagnosis of LGD on biopsies due to inter-observer variation, and therefore these current guidelines suggest the need for histologically proven LGD across at least two endoscopies before RFA. There is potential for our data to be applied to this scenario to aid a diagnosis of dysplasia based on our identified protein signature of moderate-strong epithelial cytoplasmic and nuclear HMGB1 and p53, with adjacent Foxp3+ T cell stromal infiltrate in dysplastic BO.

One limitation of this study is the inability to predict future development of progression at diagnosis as we did not perform temporal analyses in individual patients over repeat endoscopies. This is an important question and we plan to pursue this in future studies. A strength is the paired samples from dysplasia or cancer and adjacent background non-dysplastic metaplasia. We acknowledge that the potential biomarkers of oesophageal neoplastic progression have been identified from a retrospective cohort and require independent validation. However, given the small percentage of individuals with BO who progress to dysplasia or cancer, a prospective study of this nature would be large and over a long-time frame to reach statistical power.

The stromal microenvironment of a tumour is important in the progression of malignancy.^32^ Here, for the first time, we demonstrate a dynamic change in lymphocyte immune cell phenotype in BO compared with normal mucosa, and dysplastic BO. Lymphocyte signature in the background non-dysplastic BO was also associated with simultaneous diagnosis of dysplasia or cancer. We report a significantly lower number of CD20^+^ B-cells in non-dysplastic BO compared with normal epithelium, which re-emerge in dysplasia. There have been no previous studies reporting B-cell infiltrate in BO. However, in oesophageal adenocarcinoma an increased B-cell density was associated with a better survival.^49^ In a meta-analysis of 22 studies including over 2000 patients with oesophageal cancer, Zheng et al. reported that tumour infiltrating lymphocyte density was associated with better survival, with particular association seen with CD8+ and Foxp3+ cells.^50^ Infiltrating T-cells change in subtype from oesophagitis to BO to malignancy with a differential cytokine response, indicating altered immune cell function in oesophageal neoplastic progression.^51^ The mechanism and biological consequence of this dynamic stromal inflammatory infiltrate in oesophageal neoplastic progression is unknown.

The next focus will be to define the biological consequences of differential HMGB1 expression and inflammatory cell infiltrate in progressive oesophageal pathology. HMGB1 could be protective or pathogenic at a cell level.^52^ Pro-tumour effects may result from impact on the stromal inflammatory microenvironment in cancer and through effects on tumour energy metabolism, tumour progression, tissue invasion, angiogenesis and metastasis.^52^ Anti-tumour effects could be a consequence from interaction with various tumour suppressor proteins such as retinoblastoma and p53. For example, HMGB1 induces a retinoblastoma protein-dependent G1 cell-cycle arrest in breast cancer, thus functioning as a tumour suppressor protein and inducer of apoptosis.^53^ However, these cellular mechanistic studies in human tissue are challenged by a lack of access to fresh tissue given changes in clinical practice with increasing oncological focus on treatment of cancer and endoscopic resection of early lesions, with oesophagectomy rarely performed outside tertiary centres. There are also limitations and challenges in available mouse models of BO or BO cell lines that reflects pathology of the human disease. Endoscopic biopsies can be used in this circumstance but are limited in tissue yield and can be useful for stromal cell phenotyping, but there may be limitations for downstream functional studies. However, this is an advancing field and there is hope for future epithelial directed studies given the emergence of successful protocols for human BO derived epithelial organoids^54^ and single cell transcriptomics.

Here, by demonstrating emergence of cytoplasmic HMGB1, nuclear p53 and nuclear RUNX3 expression in oesophageal neoplastic progression, alongside a dynamic inflammatory cell infiltrate adjacent to BO mucosa, we offer novel mechanistic insights into the pathogenesis of BO progression to malignant transformation. This offers translational potential as a novel biomarker to predict disease progression and a potential novel pathway to target for new treatment strategies to deter malignant transformation in BO.

## Supplementary information

Supplementary data combined MD-2019-3049R2

## Data Availability

All data is published within this paper and within accompanying supporting files (indicated in text) and accessed via weblink on the journal site.

## References

[1] Cancer Research UK. Cancer statistics for the UK. www.cancerresearchuk.org/health-professional/cancer-statistics-for-the-uk. (2019).

[2] Bray F, Ferlay J, Soerjomataram I, Siegel RL, Torre LA, Jemal A (2018). Global cancer statistics 2018: GLOBOCAN estimates of incidence and mortality worldwide for 36 cancers in 185 countries. CA Cancer J. Clin..

[3] Evans RP, Mourad MM, Fisher SG, Bramhall SR (2016). Evolving management of metaplasia and dysplasia in Barrett’s epithelium. World J. Gastroenterol..

[4] Gatenby P, Caygill C, Wall C, Bhatacharjee S, Ramus J, Watson A (2014). Lifetime risk of esophageal adenocarcinoma in patients with Barrett’s esophagus. World J. Gastroenterol..

[5] Bhat S, Coleman HG, Yousef F, Johnston BT, McManus DT, Gavin AT (2011). Risk of malignant progression in Barrett’s esophagus patients: results from a large population-based study. J. Natl. Cancer Inst..

[6] Desai TK, Krishnan K, Samala N, Singh J, Cluley J, Perla S (2012). The incidence of oesophageal adenocarcinoma in non-dysplastic Barrett’s oesophagus: a meta-analysis. Gut.

[7] Al-Kasspooles MF, Hill HC, Nava HR, Smith JL, Douglass HO, Gibbs JF (2002). High-grade dysplasia within Barrett’s esophagus: controversies regarding clinical opinions and approaches. Ann. Surg Oncol..

[8] Fitzgerald RC, di Pietro M, Ragunath K, Ang Y, Kang JY, Watson P (2014). British Society of Gastroenterology guidelines on the diagnosis and management of Barrett’s oesophagus. Gut.

[9] Clemons NJ, Koh SY, Phillips WA (2014). Advances in understanding the pathogenesis of Barrett’s esophagus. Discov. Med..

[10] Konda VJA, Souza RF (2018). Biomarkers of Barrett’s esophagus: from the laboratory to clinical practice. Dig. Dis. Sci..

[11] Stachler MD, Camarda ND, Deitrick C, Kim A, Agoston AT, Odze RD (2018). Detection of mutations in Barrett’s esophagus before progression to high-grade dysplasia or adenocarcinoma. Gastroenterology.

[12] Mallick R, Patnaik SK, Wani S, Bansal A (2016). A systematic review of esophageal microRNA markers for diagnosis and monitoring of Barrett’s esophagus. Dig. Dis. Sci..

[13] Li X, Kleeman S, Coburn SB, Fumagalli C, Perner J, Jammula S (2018). Selection and application of tissue microRNAs for nonendoscopic diagnosis of Barrett’s esophagus. Gastroenterology.

[14] Shah AK, Hartel G, Brown I, Winterfold C, Na R, Le Cao K-A (2018). Evaluation of serum glycoprotein biomarker candidates for detection of esophageal adenocarcinoma and surveillance of Barrett’s esophagus. Mol. Cell Proteomics.

[15] Chan DK, Zakko L, Visrodia KH, Leggett CL, Lutzke LS, Clemens MA (2017). Breath testing for Barrett’s esophagus using exhaled volatile organic compound profiling with an electronic nose device. Gastroenterology.

[16] Blair RH, Horn AE, Pazhani Y, Grado L, Goodrich JA, Kugel JF (2016). The HMGB1 c-terminal tail regulates DNA bending. J. Mol. Biol..

[17] Lu B, Antoine DJ, Kwan K, Lundbäck P, Wähämaa H, Schierbeck H (2014). JAK/STAT1 signaling promotes HMGB1 hyperacetylation and nuclear translocation. Proc. Natl Acad. Sci. USA.

[18] Yang H, Wang H, Chavan SS, Andersson U (2015). High mobility group box protein 1 (HMGB1): the prototypical endogenous danger molecule. Mol. Med..

[19] Bertheloot D, Latz E (2017). HMGB1, IL-1α, IL-33 and S100 proteins: dual-function alarmins. Cell Mol. Immunol..

[20] Chuangui C, Peng T, Zhentao Y (2012). The expression of high mobility group box 1 is associated with lymph node metastasis and poor prognosis in esophageal squamous cell carcinoma. Pathol. Oncol. Res..

[21] Bieging KT, Mello SS, Attardi LD (2014). Unravelling mechanisms of p53-mediated tumour suppression. Nat. Rev. Cancer.

[22] Chung SM, Kao J, Hyjek E, Chen YT (2007). p53 in esophageal adenocarcinoma: a critical reassessment of mutation frequency and identification of 72Arg as the dominant allele. Int. J. Oncol..

[23] Rowell JP, Simpson KL, Stott K, Watson M, Thomas JO (2012). HMGB1-facilitated p53 DNA binding occurs via HMG-Box/p53 transactivation domain interaction, regulated by the acidic tail. Structure.

[24] Yan HX, Wu HP, Zhang HL, Ashton C, Tong C, Wu H (2013). P53 promotes inflammation-associated hepatocarcinogenesis by inducing HMGB1 release. J. Hepatol..

[25] Livesey KM, Kang R, Vernon P, Buchser W, Loughran P, Watkins SC (2012). p53/HMGB1 complexes regulate autophagy and apoptosis. Cancer Res..

[26] Ebihara T, Song C, Ryu SH, Plougastel-Douglas B, Yang L, Levanon D (2015). Runx3 specifies lineage commitment of innate lymphoid cells. Nat. Immunol..

[27] Wang Y, Qin X, Wu J, Qi B, Tao Y, Wang W (2014). Association of promoter methylation of RUNX3 gene with the development of esophageal cancer: a meta analysis. PLoS One.

[28] Schulmann K, Sterian A, Berki A, Yin J, Sato F, Xu Y (2005). Inactivation of p16, RUNX3, and HPP1 occurs early in Barrett’s-associated neoplastic progression and predicts progression risk. Oncogene.

[29] Su Z, Lu H, Jiang H, Zhu H, Li Z, Zhang P (2015). IFN-γ-producing Th17 cells bias by HMGB1-T-bet/RUNX3 axis might contribute to progression of coronary artery atherosclerosis. Atherosclerosis.

[30] Li G, Liang X, Lotze MT (2013). HMGB1: the central cytokine for all lymphoid cells. Front. Immunol..

[31] Paudel YN, Angelopoulou E, Piperi C, Balasubramaniam VRMT, Othman I, Shaikh MF (2019). Enlightening the role of high mobility group box 1 (HMGB1) in inflammation: updates on receptor signalling. Eur. J. Pharmacol..

[32] Hanahan D, Weinberg RA (2011). Hallmarks of cancer: the next generation. Cell.

[33] Bain GH, Collie-Duguid E, Murray GI, Gilbert FJ, Denison A, McKiddie F (2014). Tumour expression of leptin is associated with chemotherapy resistance and therapy-independent prognosis in gastro-oesophageal adenocarcinomas. Br. J. Cancer.

[34] Brown GT, Cash BG, Blihoghe D, Johansson P, Alnabulsi A, Murray GI (2014). The expression and prognostic significance of retinoic acid metabolising enzymes in colorectal cancer. PLoS One.

[35] McLean MH, Thomson AJ, Murray GI, Fyfe N, Hold GL, El-Omar EM (2013). Expression of neutrophil gelatinase-associated lipocalin in colorectal neoplastic progression: a marker of malignant potential?. Br. J. Cancer.

[36] Swan R, Alnabulsi A, Cash B, Alnabulsi A, Murray I (2016). Characterisation of the oxysterol metabolising enzyme pathway in mismatch repair proficient and deficient colorectal cancer. Oncotarget.

[37] McLean MH, Murray GI, Stewart KN, Norrie G, Mayer C, Hold GL (2011). The inflammatory microenvironment in colorectal neoplasia. PLoS One.

[38] Alnabulsi A, Swan R, Cash B, Alnabulsi A, Murray GI (2017). The differential expression of omega-3 and omega-6 fatty acid metabolising enzymes in colorectal cancer and its prognostic significance. Br. J. Cancer.

[39] Ross-Innes CS, Chettouh H, Achilleos A, Galeano-Dalmau N, Debiram-Beecham I, MacRae S (2017). Risk stratification of Barrett’s oesophagus using a non-endoscopic sampling method coupled with a biomarker panel: a cohort study. Lancet Gastroenterol. Hepatol..

[40] Ross-Innes CS, Debiram-Beecham I, O’Donovan M, Walker E, Varghese S, Lao-Sirieix P (2015). Evaluation of a minimally invasive cell sampling device coupled with assessment of trefoil factor 3 expression for diagnosing Barrett’s esophagus: a multi-center case–control study. PLoS Med..

[41] Chettouh H, Mowforth O, Galeano-Dalmau N, Bezawada N, Ross-Innes C, Macrae S (2018). Methylation panel is a diagnostic biomarker for Barrett’s oesophagus in endoscopic biopsies and non-endoscopic cytology specimens. Gut.

[42] El-Serag HB, Graham DY (2011). Routine polypectomy for colorectal polyps and ablation for Barrett’s esophagus are intellectually the same. Gastroenterology.

[43] Hur C, Choi SE, Rubenstein JH, Kong CY, Nishioka NS, Provenzale DT (2012). The cost-effectiveness of radiofrequency ablation for Barrett’s esophagus. Gastroenterology.

[44] Qumseya BJ, Wani S, Desai M, Qumseya A, Bain P, Sharma P (2016). Adverse events after radiofrequency ablation in patients with Barrett’s esophagus: a systematic review and meta-analysis. Clin. Gastroenterol. Hepatol..

[45] Komanduri S, Muthusamy VR, Wani S (2018). Controversies in endoscopic eradication therapy for Barrett’s esophagus. Gastroenterology.

[46] Das A, Callenberg K, Styn M, Jackson S (2016). Endoscopic ablation is a cost-effective cancer preventative therapy in patients with Barrett’s esophagus who have elevated genomic instability. Endosc. Int. Open..

[47] di Pietro M, Fitzgerald RC (2018). Revised British Society of Gastroenterology recommendation on the diagnosis and management of Barrett’s oesophagus with low-grade dysplasia. Gut.

[48] Phoa KN, van Vilsteren FGI, Weusten BLAM, Bisschops R, Schoon EJ, Ragunath K (2014). Radiofrequency ablation vs endoscopic surveillance for patients with Barrett esophagus and low-grade dysplasia. JAMA.

[49] Knief J, Reddemann K, Petrova E, Herhahn T, Wellner U, Thorns C (2016). High density of tumor-infiltrating B-lymphocytes and plasma cells signifies prolonged overall survival in adenocarcinoma of the esophagogastric junction. Anticancer Res..

[50] Zheng X, Song X, Shao Y, Xu B, Hu W, Zhou Q (2018). Prognostic role of tumor-infiltrating lymphocytes in esophagus cancer: a meta-analysis. Cell Physiol. Biochem..

[51] Kavanagh ME, Conroy MJ, Clarke NE, Gilmartin NT, O’Sullivan KE, Feighery R (2016). Impact of the inflammatory microenvironment on T-cell phenotype in the progression from reflux oesophagitis to Barrett oesophagus and oesophageal adenocarcinoma. Cancer Lett..

[52] Kang R, Zhang Q, Zeh HJ, Lotze MT, Tang D (2013). HMGB1 in cancer: good, bad, or both?. Clin. Cancer Res..

[53] Jiao Y, Wang HC, Fan SJ (2007). Growth suppression and radiosensitivity increase by HMGB1 in breast cancer. Acta Pharmacol. Sin..

[54] Liu X, Cheng Y, Abraham JM, Wang Z, Wang Z, Ke X (2018). Modeling Wnt signaling by CRISPR-Cas9 genome editing recapitulates neoplasia in human Barrett epithelial organoids. Cancer Lett..

