# Supplementary material for: Novel biomarkers for risk stratification of Barrett’s oesophagus associated neoplastic progression–epithelial HMGB1 expression and stromal lymphocytic phenotype
Source: Br J Cancer. 2019 Dec 13;122(4):545–54. doi: 10.1038/s41416-019-0685-1 (PMC7028982; doi:10.1038/s41416-019-0685-1)
Supplement: Supplementary file 1 — Supplementary data combined MD-2019-3049R2 [file 41416_2019_685_MOESM1_ESM.pdf]

Table S1: Patient characteristics and relationship between clinicopathological data and overall survival			
	**Number of Patients (%)**	**2**	**p**
**Age at diagnosis***			
<65	17 (30%)	0.515	0.473
>65	40 (70%)		
**Gender**			
Male	12 (21%)	0.085	0.771
Female	46 (79%)		
**pT Stage**			
T1	12 (21%)	4.801	0.091
T2	13 (22%)		
T3	33 (57%)		
T4	0 (0%)		
**N Stage**			
N0	29 (50%)	5.279	0.071
N1	28 (48%)		
N2	1 (2%)		
N3	0 (0%)		
**M Stage**			
0	58 (100%)	-	-
1	0 (0%)		
**Stage I-IV**			
I (a/b)	13 (22%)	5.113	0.078
II (a/b)	19 (33%)		
III	26 (45%)		
IV	0 (0%)		
**Chemotherapy Status****			
Received Chemotherapy	7 (13%)	2.003	0.157
Did not receive Chemotherapy	46 (87%)		
**Mandard Regression Score*****			
1	0 (0%)	4.428	0.219
2	2 (6%)		
3	6 (17%)		
4	16 (46%)		
5	11 (31%)		
**30 Day Surgical Mortality***			
Alive >30 days	48 (84%)	90.767	**<0.001**
Alive <30 days	9 (16%)		
No data are available for *1, **5 and ***23 patients.			

Table 2: Characteristics of the antibodies used for immunohistochemistry

Antibody target	Antibody type	Antigen retrieval buffer	Dilution	Positive control	Supplier	Code	Isotype, clone
HMGB1	rabbit monoclonal	citrate	1:400	colorectal cancer	abcam	ab79823	IgG, EPR3507
p53	mouse monoclonal	EDTA	1:250	colorectal cancer	abcam	ab1101	IgG2a, DO-1
RUNX3	mouse monoclonal	EDTA	1:500	colorectal cancer	abcam	ab40278	IgG1, R3-5G4
CD20+ B-cells	mouse monoclonal	citrate	1:600	tonsil	Agilent Technologies	M 075529-2	IgG2a, L26
CD4+ T-cells	mouse monoclonal	EDTA	1:500	tonsil	abcam	ab133616	IgG, EPR6855
CD8+ T-cells	mouse monoclonal	EDTA	1:150	tonsil	abcam	ab17147	IgG1, 144B
Foxp3+ T-cells (Tregs)	mouse monoclonal	EDTA	1:200	tonsil	abcam	ab20034	IgG1, 236A/E7

Note: Citrate buffer at pH 6 and ethylenediaminetetraacetic acid (EDTA) at pH 7.8

Table S3: Association between HMGB1 and histological diagnosis; extended analysis

	Absent v weak v moderate v strong				Absent v weak, moderate and strong				Absent and weak v moderate and				Strong v absent, weak and moderate					
	Nucleus		Cytoplasm		Nucleus		Cytoplasm		Nucleus		Cytoplasm		Nucleus		Cytoplasm			
**Comparrisons**	**χ2**	**p**	**χ2**	**p**	**χ2**	**p**	**χ2**	**p**	**χ2**	**p**	**χ2**	**p**	**χ2**	**p**	**χ2**	**p**	**Nuclear Relationship**	**Cytoplasmic Relationship**
**Pre-malignant comparisons**																		
NO v NG	4.811	0.078	2.320	0.652	*	*	-	0.543	-	1.000	-	1.000	-	0.052	-	1.000	-	-
NO v ND BO	18.257	**<0.001**	32.086	**<0.001**	-	1.000	-	**<0.001**	-	**0.019**	-	**<0.001**	-	**<0.001**	-	0.338	NO < ND BO	NO < ND BO
NO v D BO	0.885	0.303	24.723	**<0.001**	*	*	-	**0.001**	-	0.579	-	**<0.001**	-	0.674	-	**<0.001**	-	NO < D BO
NO v ND BO p-dys	1.052	0.270	19.497	**<0.001**	*	*	-	**0.001**	-	0.568	-	**<0.001**	-	0.669	-	0.183	-	NO < ND BO p-dys
NO v ND BO p- OAC	1.452	0.283	3.109	0.437	*	*	-	0.420	-	1.000	-	0.295	-	0.367	-	0.333	-	-
NG v ND BO	8.339	**0.028**	42.153	**<0.001**	-	1.000	-	**<0.001**	-	0.018	-	**<0.001**	-	**0.028**	-	0.675	NG < ND BO	NG < ND BO
NG v D BO	3.278	0.164	28.841	**<0.001**	*	*	-	**0.003**	-	0.622	-	**<0.001**	-	0.318	-	**0.001**	-	NG < D BO
NG v ND BO p-dys	2.930	0.268	24.883	**<0.001**	*	*	-	**0.003**	-	0.609	-	**<0.001**	-	0.317	-	0.289	-	NG < ND BO p-dys
NG v ND BO p-OAC	1.153	0.520	3.716	0.264	*	*	-	0.704	-	1.000	-	0.102	-	0.697	-	0.465	-	-
ND BO v D BO	10.745	**0.008**	13.196	**0.002**	-	1.000	-	1.000	-	0.137	-	0.177	-	**0.002**	-	**<0.001**	ND BO < D BO	ND < D BO
ND BO v ND BO p-dys	9.417	**0.017**	0.872	0.887	-	1.000	-	1.000	-	0.211	-	0.509	-	**0.004**	-	0.631	ND BO < ND BO p-dys	-
ND BO v ND BO p-OAC	5.625	0.164	10.397	**0.008**	-	1.000	-	**0.015**	-	0.261	-	**0.015**	-	**0.024**	-	1.000	ND BO < ND BO p-OAC	ND BO > ND BO p-OAC
D BO v ND BO p-dys	0.269	1.000	4.839	0.072	*	*	-	*	-	1.000	-	0.598	-	1.000	-	**0.050**	-	D BO > ND BO p-dys
D BO v ND BO p-OAC	0.625	0.822	9.591	**0.015**	*	*	-	**0.042**	-	1.000	-	**0.004**	-	1.000	-	0.080	-	D BO > ND BO p-OAC
ND BO p-dys v ND BO p-OAC	0.542	1.000	7.680	**0.027**	*	*	-	**0.047**	-	1.000	-	**0.023**	-	1.000	-	1.000	-	ND BO p-dys > ND BO p-OAC
**Oesophageal adenocarcinoma**																		
OAC v NO	17.973	**<0.001**	15.788	**0.001**	-	1.000	-	**<0.001**	-	0.116	-	**0.002**	-	**<0.001**	-	**0.040**	OAC < NO	OAC > NO
OAC v NG	5.630	0.111	17.701	**<0.001**	-	0.581	-	**0.004**	-	0.099	-	**<0.001**	-	**0.030**	-	**0.044**	OAC < NG	OAC > NG
OAC v ND BO	4.023	0.257	24.814	**<0.001**	-	0.392	-	**0.037**	-	0.232	-	**0.001**	-	1.000	-	**0.039**	-	OAC < ND BO
OAC v D BO	10.931	**0.008**	10.727	**0.008**	-	1.000	-	0.209	-	0.514	-	**0.002**	-	**0.001**	-	**0.022**	OAC < D BO	OAC < D BO
OAC v ND BO p-dys	9.543	**0.017**	8.844	**0.020**	-	1.000	-	0.209	-	0.515	-	**0.019**	-	**0.002**	-	0.731	OAC < ND BO p-dys	OAC < ND BO p-dys
OAC v ND BO p-OAC	4.782	0.142	2.245	0.563	-	1.000	-	0.152	-	0.682	-	0.490	-	**0.024**	-	0.682	OAC < ND BO p-OAC	-

Note. *no statistics are computed because one variable is a constant. - no Chi square value is provided as test was with a 2X2 contingency table

Table S4: Association between p53 and histological diagnosis; extended analysis																		
	Absent v weak v moderate v strong				Absent v weak, moderate and strong				Absent and weak v moderate and strong				Strong v absent, weak and moderate					
	Nucleus		Cytoplasm		Nucleus		Cytoplasm		Nucleus		Cytoplasm		Nucleus		Cytoplasm			
Comparrisons	χ2	p	χ2	p	χ2	p	χ2	p	χ2	p	χ2	p	χ2	p	χ2	p	Nuclear Relationship	Cytoplasmic Relationship
**Pre-malignant comparisons**																		
NO v NG	2.975	0.139	-	0.278	-	0.139	-	0.278	-	0.375	-	*	-	0.375	*	*	-	-
NO v ND BO	12.269	**0.003**	9.986	**0.004**	-	**0.003**	-	**0.001**	-	1.000	-	0.584	-	0.310	*	*	NO < ND BO	NO < ND BO
NO v D BO	20.483	**<0.001**	27.289	**<0.001**	-	**<0.001**	-	**<0.001**	-	**<0.001**	-	**<0.001**	-	**0.002**	-	**0.002**	NO < D BO	NO < D BO
NO v ND BO p-dys	8.561	**0.010**	25.755	**<0.001**	-	**0.009**	-	**<0.001**	-	**0.014**	-	**0.002**	-	**0.014**	-	**0.042**	NO < ND BO p-dys	NO < ND BO p-dys
NO v ND BO p- OAC	1.881	0.372	*	*	-	0.670	*	*	-	1.000	*	*	-	1.000	*	*	-	-
NG v ND BO	28.200	**<0.001**	5.391	0.057	-	**<0.001**	-	**0.028**	-	0.187	-	0.333	-	1.000	*	*	NG < ND BO	NG < ND BO
NG v D BO	36.226	**<0.001**	28.741	**<0.001**	-	**<0.001**	-	**<0.001**	-	**<0.001**	-	**<0.001**	-	**<0.001**	-	**<0.001**	NG < D BO	NG < D BO
NG v ND BO p-dys	20.509	**<0.001**	24.677	**<0.001**	-	**<0.001**	-	**<0.001**	-	**<0.001**	-	**<0.001**	-	**<0.001**	-	**0.012**	NG < ND BO p-dys	NG < ND BO p-dys
NG v ND BO p-OAC	-	**0.038**	-	0.278	-	**0.038**	-	0.278	*	*	*	*	*	*	*	*	NG < ND BO p-OAC	-
ND BO v D BO	41.842	**<0.001**	35.538	**<0.001**	-	**0.031**	-	**<0.001**	-	**<0.001**	-	**<0.001**	-	**<0.001**	-	**<0.001**	ND BO < D BO	ND BO < D BO
ND BO v ND BO p-dys	23.019	**<0.001**	23.548	**<0.001**	-	0.762	-	**0.001**	-	**0.001**	-	**0.001**	-	**<0.001**	-	**<0.001**	ND BO < ND BO p-dys	ND BO < ND BO p-dys
ND BO v ND BO p-OAC	4.747	0.186	8.468	**0.010**	-	**0.035**	-	**0.003**	-	0.588	-	0.585	-	1.000	*	*	ND BO > ND BO p-OAC	ND BO > ND BO p-OAC
D BO v ND BO p-dys	5.679	0.140	2.164	0.723	-	0.169	-	1.000	-	**0.050**	-	0.462	-	0.462	-	0.264	D BO > ND BO p-dys	-
D BO v ND BO p-OAC	21.732	**<0.001**	24.793	**<0.001**	-	**0.001**	-	**<0.001**	-	**<0.001**	-	**<0.001**	-	**<0.001**	-	**0.002**	D BO > ND BO p-OAC	D BO > ND BO p-OAC
ND BO p-dys v ND BO p-OAC	9.312	**0.010**	23.380	**<0.001**	-	0.057	-	**<0.001**	-	**0.006**	-	**0.006**	-	**0.006**	-	0.098	ND BO p-dys > ND BO p-OAC	ND BO p-dys > ND BO p-OAC
**Oesophageal adenocarcinoma**																		
OAC v NO	9.671	**0.014**	0.832	1.000	-	**0.005**	-	1.000		**0.002**	-	1.000	-	**0.019**	*	*	OAC > NO	-
OAC v NG	27.987	**<0.001**	4.877	0.102	-	**<0.001**	-	0.070		**<0.001**	-	1.000	-	**<0.001**	*	*	OAC > NG	-
OAC v ND BO	63.012	**<0.001**	39.655	**<0.001**	-	0.652	-	**<0.001**		**<0.001**	-	**0.013**	-	**0.001**	*	*	OAC > ND BO	OAC < ND BO
OAC v D BO	8.717	**0.019**	64.602	**<0.001**	-	**0.010**	-	**<0.001**		**0.006**	-	**<0.001**	-	0.051	-	**<0.001**	OAC < D BO	OAC < D BO
OAC v ND BO p-dys	2.834	0.416	57.393	**<0.001**	-	0.565	-	**<0.001**		1.000	-	**<0.001**	-	0.404	-	**<0.001**	-	OAC < ND BO p-dys
OAC v ND BO p-OAC	12.355	**0.003**	0.877	1.000	-	0.073	-	1.000		**0.001**	-	1.000	-	**0.004**	*	*	OAC > ND BO p-dys	-
Note. *no statistics are computed because one variable is a constant. - no Chi square value is provided as test was with a 2X2 contingency table																		

Table S5: Association between RUNX3 and histological diagnosis; extended analysis

	Absent v weak v moderate v strong				Absent v weak, moderate and strong				Absent and weak v moderate and strong				Strong v absent, weak and moderate					
	Nucleus		Cytoplasm		Nucleus		Cytoplasm		Nucleus		Cytoplasm		Nucleus		Cytoplasm			
Comparrisons	$\chi 2$	p	$\chi 2$	p	$\chi 2$	p	$\chi 2$	p	$\chi 2$	p	$\chi 2$	p	$\chi 2$	p	$\chi 2$	p	Nuclear Relationship	Cytoplasmic Relationship
**Pre-malignant comparisons**																		
NO v NG	*	*	-	1.000	*	*	-	1.000	*	*	*	*	*	*	*	*	-	-
NO v ND BO	-	1.000	*	*	-	1.000	*	*	*	*	*	*	*	*	*	*	-	-
NO v D BO	-	**0.013**	-	0.455	-	**0.013**	-	0.455	*	*	*	*	*	*	*	*	NO < D BO	-
NO v ND BO p-dys	-	**0.010**	-	**0.028**	-	**0.010**	-	**0.028**	*	*	*	*	*	*	*	*	NO < ND BO p-dys	NO < ND BO p-dys
NO v ND BO p- OAC	*	*	*	*	*	*	*	*	*	*	*	*	*	*	*	*	-	-
NG v ND BO	-	1.000	-	0.276	-	1.000	-	0.276	*	*	*	*	*	*	*	*	-	-
NG v D BO	-	**0.004**	-	1.000	-	**0.004**	-	1.000	*	*	*	*	*	*	*	*	NG < D BO	
NG v ND BO p-dys	-	**0.003**	-	**0.039**	-	**0.003**	-	**0.039**	*	*	*	*	*	*	*	*	NG < ND BO p-dys	NG < ND BO p-dys
NG v ND BO p-OAC	*	*	-	1.000	*	*	-	1.000	*	*	*	*	*	*	*	*	-	-
ND BO v D BO	-	**0.001**	-	0.174	-	**0.001**	-	0.174	*	*	*	*	*	*	*	*	ND BO < D BO	-
ND BO v ND BO p-dys	-	**0.001**	-	**<0.001**	-	**0.001**	-	**<0.001**	*	*	*	*	*	*	*	*	ND BO < ND BO p-dys	ND BO < ND BO p-dys
ND BO v ND BO p-OAC	-	1.000	*	*	-	1.000	*	*	*	*	*	*	*	*	*	*	-	-
D BO v ND BO p-dys	-	1.000	-	0.169	-	1.000	-	0.169	*	*	*	*	*	*	*	*	-	-
D BO v ND BO p-OAC	-	0.053	-	1.000	-	0.053	-	1.000	*	*	*	*	*	*	*	*	-	-
ND BO p-dys v ND BO p-OAC	-	**0.046**	-	0.105	-	**0.046**	-	0.105	*	*	*	*	*	*	*	*	ND BO p-dys > ND BO p-OAC	-
**Oesophageal adenocarcinoma**																		
OAC v NO	0.342	1.000	*	*	*	*	*	*	-	1.000	*	*	*	*	*	*	-	-
OAC v NG	0.407	1.000	-	1.000	-	0.586	-	0.185	-	1.000	*	*	*	*	*	*	-	-
OAC v ND BO	1.789	0.416	*	*	-	1.000	*	*	-	0.294	*	*	*	*	*	*	-	-
OAC v D BO	15.943	**<0.001**	-	0.112	-	**0.002**	-	0.112	-	1.000	*	*	*	*	*	*	OAC < D BO	-
OAC v ND BO p-dys	16.708	**<0.001**	-	**<0.001**	-	**0.001**	-	**<0.001**	-	1.000	*	*	*	*	*	*	OAC < ND BO p-dys	OAC < ND BO p-dys
OAC v ND BO p-OAC	0.586	1.000	*	*	-	1.000	*	*	-	1.000	*	*	*	*	*	*	-	-

Note: *no statistics are computed because one variable is a constant. - no Chi square value is provided as test was with a 2X2 contingency table

Table S6: Association between HMGB1, p53 and RUNX3 protein expression	0 v 1 v 2 v 3		0 v 1, 2, 3	0, 1 v 2 3	3 v 0, 1, 2
	χ^2	p	p	p	p
**All Oesophageal Tissue (normal, Barrett's, dysplasia and cancer)**					
HMGB1 Nuclear vs. HMGB1 Cytoplasmic	19.473	**0.021**	0.598	0.098	0.482
HMGB1 Nuclear vs. p53 Nuclear	7.655	0.569	0.412	0.432	0.767
HMGB1 Nuclear vs. RUNX3 Nuclear	10.391	0.109	1	0.34	-
p53 Nuclear vs. p53 Cytoplasmic	81.159	**<0.001**	**<0.001**	**<0.001**	**<0.001**
p53 Nuclear vs. RUNX3 Nuclear	22.871	**0.001**	**0.002**	**0.024**	-
RUNX3 Nuclear vs. RUNX3 Cytoplasmic	85.269	**<0.001**	**<0.001**	-	-
HMGB1 Cytoplasmic vs. p53 Cytoplasmic	25.13	**0.003**	**0.004**	**0.013**	**0.018**
HMGB1 Cytoplasmic vs. RUNX3 Cytoplasmic	3.308	0.347	1	-	-
p53 Cytoplasmic vs. RUNX3 Cytoplasmic	44.32	**<0.001**	**0.001**	-	-
HMGB1 Cytoplasmic vs. p53 Nuclear	15.768	0.072	**0.002**	0.493	0.127
HMGB1 Cytoplasmic vs. RUNX3 Nuclear	8.336	0.205	0.085	0.688	-
**Normal Oesophagus**					
HMGB1 Nuclear vs. HMGB1 Cytoplasmic	2.88	0.578	-	1	-
HMGB1 Nuclear vs. p53 Nuclear	0.294	0.863	-	-	1
HMGB1 Nuclear vs. RUNX3 Nuclear	-	-	-	-	-
p53 Nuclear vs. p53 Cytoplasmic	-	-	-	-	-
p53 Nuclear vs. RUNX3 Nuclear	-	-	-	-	-
RUNX3 Nuclear vs. RUNX3 Cytoplasmic	-	-	-	-	-
HMGB1 Cytoplasmic vs. p53 Cytoplasmic	-	-	-	-	-
HMGB1 Cytoplasmic vs. RUNX3 Cytoplasmic	-	-	-	-	-
p53 Cytoplasmic vs. RUNX3 Cytoplasmic	-	-	-	-	-
HMGB1 Cytoplasmic vs. p53 Nuclear	5.091	0.264	0.209	-	-
HMGB1 Cytoplasmic vs. RUNX3 Nuclear	-	-	-	-	-
**All Barrett's oesophagus**					
HMGB1 Nuclear vs. HMGB1 Cytoplasmic	25.055	**0.003**	1	**0.006**	**0.008**
HMGB1 Nuclear vs. p53 Nuclear	16.647	0.055	1	0.136	**0.001**
HMGB1 Nuclear vs. RUNX3 Nuclear	7.601	0.055	1	-	-
p53 Nuclear vs. p53 Cytoplasmic	87.008	**<0.001**	**<0.001**	**<0.001**	**<0.001**
p53 Nuclear vs. RUNX3 Nuclear	35.004	**<0.001**	0.053	-	-
RUNX3 Nuclear vs. RUNX3 Cytoplasmic	43.196	**<0.001**	**<0.001**	-	-
HMGB1 Cytoplasmic vs. p53 Cytoplasmic	16.218	0.062	1	0.778	**0.004**
HMGB1 Cytoplasmic vs. RUNX3 Cytoplasmic	1.134	0.72	1	-	-
p53 Cytoplasmic vs. RUNX3 Cytoplasmic	21.534	**<0.001**	0.057	-	-
HMGB1 Cytoplasmic vs. p53 Nuclear	24.531	**0.005**	0.112	**0.036**	0.074
HMGB1 Cytoplasmic vs. RUNX3 Nuclear	0.583	1	1	-	-
**All Non-dysplastic Barrett's oesophagus**					
HMGB1 Nuclear vs. HMGB1 Cytoplasmic	22.723	**0.007**	1	**0.001**	0.379
HMGB1 Nuclear vs. p53 Nuclear	9.588	0.385	1	1	1
HMGB1 Nuclear vs. RUNX3 Nuclear	1.08	0.782	1	-	-
p53 Nuclear vs. p53 Cytoplasmic	25.891	**<0.001**	**<0.001**	0.096	-
p53 Nuclear vs. RUNX3 Nuclear	6.653	0.084	1	-	-
RUNX3 Nuclear vs. RUNX3 Cytoplasmic	-	-	-	-	-
HMGB1 Cytoplasmic vs. p53 Cytoplasmic	8.159	0.227	0.166	0.134	-
HMGB1 Cytoplasmic vs. RUNX3 Cytoplasmic	-	-	-	-	-
p53 Cytoplasmic vs. RUNX3 Cytoplasmic	-	-	-	-	-
HMGB1 Cytoplasmic vs. p53 Nuclear	12.271	0.192	1	1	1

HMGB1 Cytoplasmic vs. RUNX3 Nuclear	3.855	0.278	1	-	-
**Dysplastic Barrett's oesophagus**					
HMGB1 Nuclear vs. HMGB1 Cytoplasmic	3.949	0.413	-	1	0.282
HMGB1 Nuclear vs. p53 Nuclear	9.909	0.129	-	1	0.56
HMGB1 Nuclear vs. RUNX3 Nuclear	5.182	0.075	-	-	-
p53 Nuclear vs. p53 Cytoplasmic	19.375	**0.022**	1	1	**0.007**
p53 Nuclear vs. RUNX3 Nuclear	3.75	0.29	1	-	-
RUNX3 Nuclear vs. RUNX3 Cytoplasmic	2.143	0.143	0.333	-	-
HMGB1 Cytoplasmic vs. p53 Cytoplasmic	8.984	0.174	-	1	0.619
HMGB1 Cytoplasmic vs. RUNX3 Cytoplasmic	15	**<0.001**	-	-	-
p53 Cytoplasmic vs. RUNX3 Cytoplasmic	6.964	0.073	1	-	-
HMGB1 Cytoplasmic vs. p53 Nuclear	3.833	0.699	-	1	0.608
HMGB1 Cytoplasmic vs. RUNX3 Nuclear	4.313	0.116	-	-	-
**Non-dysplastic Barrett's oesophagus in patients who have progressed to dysplasia**					
HMGB1 Nuclear vs. HMGB1 Cytoplasmic	3.36	0.499	-	1	1
HMGB1 Nuclear vs. p53 Nuclear	6.8	0.147	-	0.462	0.559
HMGB1 Nuclear vs. RUNX3 Nuclear	3.111	0.211	-	-	-
p53 Nuclear vs. p53 Cytoplasmic	5.875	0.437	0.286	0.286	0.559
p53 Nuclear vs. RUNX3 Nuclear	3.63	0.163	0.221	-	-
RUNX3 Nuclear vs. RUNX3 Cytoplasmic	10.08	**0.001**	**0.005**	-	-
HMGB1 Cytoplasmic vs. p53 Cytoplasmic	4.9	0.557	-	0.462	0.505
HMGB1 Cytoplasmic vs. RUNX3 Cytoplasmic	2.24	0.326	-	-	-
p53 Cytoplasmic vs. RUNX3 Cytoplasmic	5.833	0.12	1	-	-
HMGB1 Cytoplasmic vs. p53 Nuclear	12.367	**0.015**	-	-	0.462
HMGB1 Cytoplasmic vs. RUNX3 Nuclear	1.369	0.504	-	-	-
**Non-dysplastic Barrett's oesophagus in patients who have progressed to adenocarcinoma**					
HMGB1 Nuclear vs. HMGB1 Cytoplasmic	4	0.677	-	1	1
HMGB1 Nuclear vs. p53 Nuclear	0.875	0.646	-	-	-
HMGB1 Nuclear vs. RUNX3 Nuclear	-	-	-	-	-
p53 Nuclear vs. p53 Cytoplasmic	-	-	-	-	-
p53 Nuclear vs. RUNX3 Nuclear	-	-	-	-	-
RUNX3 Nuclear vs. RUNX3 Cytoplasmic	-	-	-	-	-
HMGB1 Cytoplasmic vs. p53 Cytoplasmic	-	-	-	-	-
HMGB1 Cytoplasmic vs. RUNX3 Cytoplasmic	-	-	-	-	-
p53 Cytoplasmic vs. RUNX3 Cytoplasmic	-	-	-	-	-
HMGB1 Cytoplasmic vs. p53 Nuclear	7	0.072	1	-	-
HMGB1 Cytoplasmic vs. RUNX3 Nuclear	-	-	-	-	-
**Oesophageal Adenocarcinoma**					
HMGB1 Nuclear vs. HMGB1 Cytoplasmic	20.312	**0.016**	1	0.815	0.787
HMGB1 Nuclear vs. p53 Nuclear	10.197	0.335	0.293	0.816	0.491
HMGB1 Nuclear vs. RUNX3 Nuclear	3.289	0.772	1	1	-
p53 Nuclear vs. p53 Cytoplasmic	47.82	**<0.001**	**0.08**	1	-
p53 Nuclear vs. RUNX3 Nuclear	16.142	**0.013**	0.647	0.612	-
RUNX3 Nuclear vs. RUNX3 Cytoplasmic	-	-	-	-	-
HMGB1 Cytoplasmic vs. p53 Cytoplasmic	4.717	0.581	0.589	1	-
HMGB1 Cytoplasmic vs. RUNX3 Cytoplasmic	-	-	-	-	-
p53 Cytoplasmic vs. RUNX3 Cytoplasmic	-	-	-	-	-
HMGB1 Cytoplasmic vs. p53 Nuclear	5.664	0.773	0.37	0.242	1
HMGB1 Cytoplasmic vs. RUNX3 Nuclear	12.936	**0.044**	1	1	-
Note: - no Chi square value is provided as test was with a 2X2 contingency table					

Table S7: CD20+, CD4+, CD8+ and FOXP3+ lymphocytes in pre-malignant oesophageal neoplastic progression				
Histology	Number of samples	Median number of positive cells (SD)	25th percentile	75th percentile
**$CD20^+$ B-cells**				
NO	10	14 (23.300)	5.75	45.75
NG	15	45 (38.221)	34	71
ND BO	70	0 (1.326)	0	0
D BO	13	0 (5.294)	0	7
ND BO p-dys	14	0 (4.480)	0	3.25
ND BO p-OAC	14	2 (15.745)	0	15.5
**$CD4^+$ T-cells**				
NO	18	80 (66.261)	53.5	141.75
NG	18	57 (46.390)	26.75	92.5
ND BO	56	21 (21.186)	11	35.25
D BO	15	26 (39.071)	14	53
ND BO p-dys	12	20.50 (34.630)	12.25	68.25
ND BO p-OAC	11	35 (27.359)	31	64
**$CD8^+$ T-cells**				
NO	13	80 (55.073)	49.5	114.5
NG	33	89 (53.242)	65.5	102.5
ND BO	61	16 (17.787)	7	34.5
D BO	15	31 (66.966)	17	89
ND BO p-dys	14	33.50 (41.589)	7.5	61.5
ND BO p-OAC	22	33 (27.399)	11.75	66.25
**$Foxp3^+$ T-cells (Tregs)**				
NO	19	9 (16.305)	1	30
NG	16	5.50 (11.615)	2	10
ND BO	57	9 (10.348)	3	17
D BO	15	36 (34.117)	22	68
ND BO p-dys	13	11 (26.878)	3	33
ND BO p-OAC	16	8 (16.564)	1	28.75

Table S8: Association between lymphocyte populations and histological cell types.

Comparisons	Mann Whitney-U	p	Relationship
**CD20$^+$ B-cells**			
NO v NG	41.5	0.063	-
NO v ND BO	15	**<0.001**	NO > ND BO
NO v D BO	19.5	**0.004**	NO > D BO
NO v ND BO p-dys	14.5	**0.001**	NO > ND BO p-dys
NO v ND BO p-OAC	32	**0.025**	NO > ND BO p-OAC
NG v ND BO	48	**<0.001**	NG > ND BO
NG v D BO	15.5	**<0.001**	NG > D BO
NG v ND BO p-dys	14.5	**<0.001**	NG > ND BO p-dys
NG v ND BO p-OAC	28	**<0.001**	NG > ND BO p-OAC
ND BO v D BO	289	**0.003**	ND BO < D BO
ND BO v ND BO p-dys	374.5	**0.038**	ND BO < ND BO p-dys
ND BO v ND BO p-OAC	231	**<0.001**	ND BO < ND BO p-OAC
D BO v ND BO p-dys	78	0.478	-
D BO v ND BO p-OAC	76	0.445	-
ND BO p-dys v ND BO p-OAC	70.5	0.176	-
**CD4$^+$ T-cells**			
NO v NG	110	0.1	-
NO v ND BO	11713	**<0.001**	NO > ND BO
NO v D BO	54	**0.003**	NO > D BO
NO v ND BO p-dys	42.5	**0.006**	NO > ND BO p-dys
NO v ND BO p-OAC	50.5	**0.029**	NO > ND BO p-OAC
NG v ND BO	219.5	**<0.001**	NG > ND BO
NG v D BO	82.5	0.058	-
NG v ND BO p-dys	60.5	**0.044**	NG > ND BO p-dys
NG v ND BO p-OAC	83	0.472	-
ND BO v D BO	364	0.43	-
ND BO v ND BO p-dys	303	0.595	-
ND BO v ND BO p-OAC	131	**0.003**	ND BO < ND BO p-OAC
D BO v ND BO p-dys	83.5	0.751	-
D BO v ND BO p-OAC	54	0.138	-
ND BO p-dys v ND BO p-OAC	35	0.056	-
**CD8$^+$ T-cells**			
NO v NG	187	0.502	-
NO v ND BO	27.5	**<0.001**	NO > ND BO
NO v D BO	55.5	0.053	-
NO v ND BO p-dys	30	**0.003**	NO > ND BO p-dys
NO v ND BO p-OAC	42	**0.001**	NO > ND BO p-OAC
NG v ND BO	61.5	**<0.001**	NG > ND BO
NG v D BO	125	**0.007**	NG > D BO
NG v ND BO p-dys	67	**<0.001**	NG > ND BO p-dys
NG v ND BO p-OAC	83	**<0.001**	NG > ND BO p-OAC

ND BO v D BO	265	**0.012**	ND BO < D BO
ND BO v ND BO p-dys	311.5	0.116	-
ND BO v ND BO p-OAC	432	**0.014**	ND BO < ND BO p-OAC
D BO v ND BO p-dys	90	0.513	-
D BO v ND BO p-OAC	139	0.421	-
ND BO p-dys v ND BO p-OAC	147	0.82	-
**Foxp3^{+} T-cells (Tregs)**			
NO v NG	119.5	0.28	-
NO v ND BO	515	0.75	-
NO v D BO	54.5	**0.002**	NO < D BO
NO v ND BO p-dys	111.5	0.644	-
NO v ND BO p-OAC	151	0.974	-
NG v ND BO	356	0.181	-
NG v D BO	24	**<0.001**	NG < D BO
NG v ND BO p-dys	72	0.159	-
NG v ND BO p-OAC	108	0.45	-
ND BO v D BO	108.5	**<0.001**	ND BO < D BO
ND BO v ND BO p-dys	310.5	0.364	-
ND BO v ND BO p-OAC	452	0.957	-
D BO v ND BO p-dys	48	**0.023**	D BO > ND BO p-dys
D BO v ND BO p-OAC	44	**0.003**	D BO > ND BO p-OAC
ND BO p-dys v ND BO p-OAC	86	0.429	-

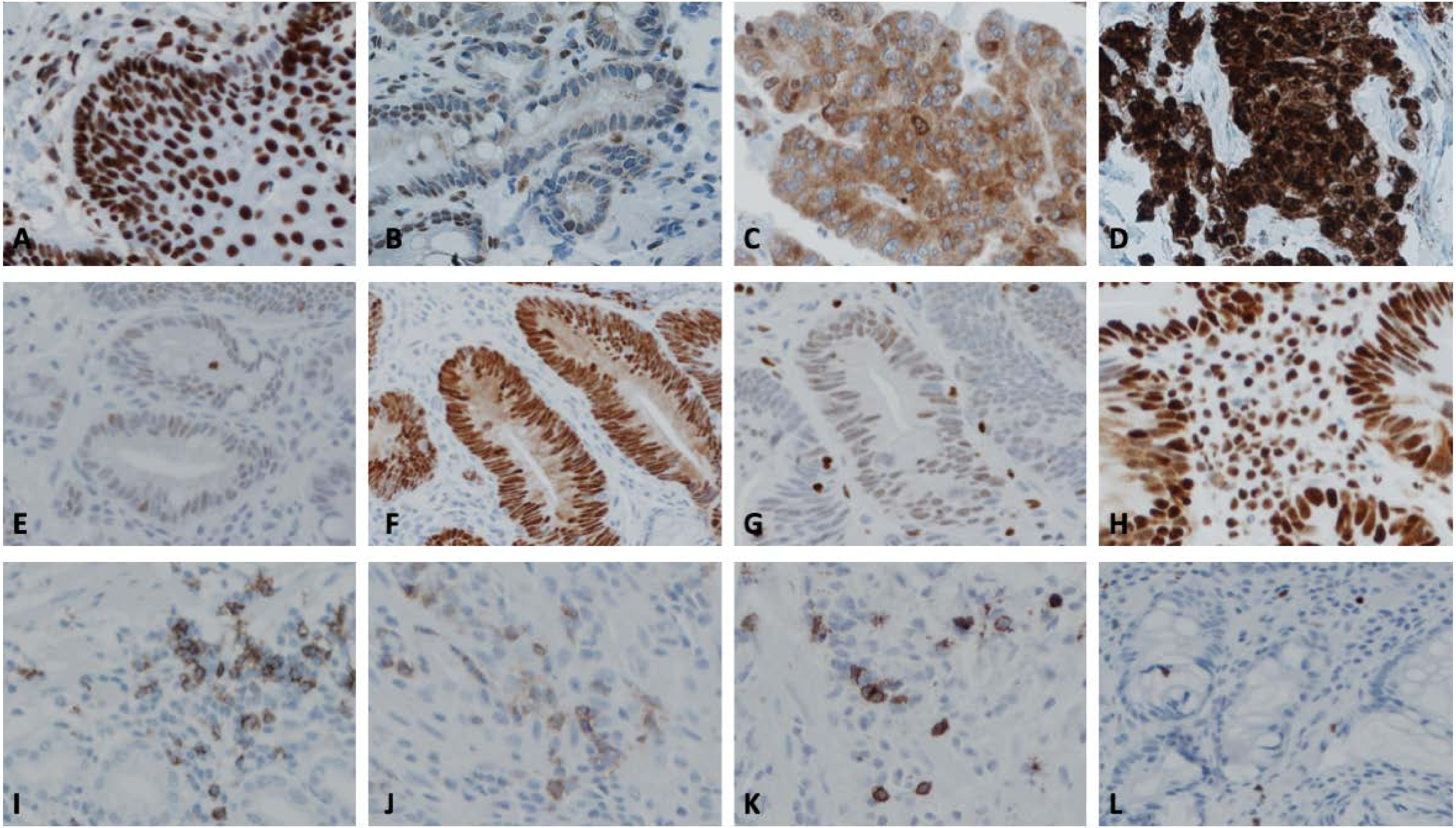
A
B
C
D
E
F
G
H
I
J
K
L

Novel biomarkers for risk stratification of Barrett's oesophagus associated neoplastic progression - epithelial HMGB1 expression and stromal lymphocytic phenotype

Figure legend Figure S1

Representative high power field photomicrographs representing (A) strong nuclear and absent cytoplasmic HMGB1 expression in normal oesophageal epithelium, (B) absent nuclear and weak cytoplasmic HMGB1 expression in non-dysplastic BO epithelium, (C) absent nuclear and moderate cytoplasmic HMGB1 expression in OAC, (D) strong nuclear and strong cytoplasmic HMGB1 expression in OAC, (E) weak nuclear p53 in non-dysplastic BO epithelium, (F) strong nuclear and moderate cytoplasmic p53 expression in dysplastic BO epithelium, (G) weak nuclear and absent cytoplasmic RUNX3 expression in dysplastic BO epithelium, (H) ubiquitous HMGB1 expression in stromal lymphocyte populations in dysplastic BO biopsy, (I) CD20 positive lymphocytes, (J) CD8 positive lymphocytes, (K) CD4 positive lymphocytes and (L) FOXP3 positive lymphocytes.
